# Recovery of bioactive protein from bacterial inclusion bodies using trifluoroethanol as solubilization agent

**DOI:** 10.1186/s12934-016-0504-9

**Published:** 2016-06-08

**Authors:** Vaibhav Upadhyay, Anupam Singh, Divya Jha, Akansha Singh, Amulya K. Panda

**Affiliations:** Product Development Cell, National Institute of Immunology, Aruna Asaf Ali Marg, New Delhi, 110 067 India

**Keywords:** Inclusion bodies, Trifluoroethanol, Human growth hormone, Mild solubilization, Refolding

## Abstract

**Background:**

Formation of inclusion bodies poses a major hurdle in recovery of bioactive recombinant protein from *Escherichia coli*. Urea and guanidine hydrochloride have routinely been used to solubilize inclusion body proteins, but many times result in poor recovery of bioactive protein. High pH buffers, detergents and organic solvents like *n*-propanol have been successfully used as mild solubilization agents for high throughput recovery of bioactive protein from bacterial inclusion bodies. These mild solubilization agents preserve native-like secondary structures of proteins in inclusion body aggregates and result in improved recovery of bioactive protein as compared to conventional solubilization agents. Here we demonstrate solubilization of human growth hormone inclusion body aggregates using 30 % trifluoroethanol in presence of 3 M urea and its refolding into bioactive form.

**Results:**

Human growth hormone was expressed in *E. coli* M15 (pREP) cells in the form of inclusion bodies. Different concentrations of trifluoroethanol with or without addition of low concentration (3 M) of urea were used for solubilization of inclusion body aggregates. Thirty percent trifluoroethanol in combination with 3 M urea was found to be suitable for efficient solubilization of human growth hormone inclusion bodies. Solubilized protein was refolded by dilution and purified by anion exchange and size exclusion chromatography. Purified protein was analyzed for secondary and tertiary structure using different spectroscopic tools and was found to be bioactive by cell proliferation assay. To understand the mechanism of action of trifluoroethanol, secondary and tertiary structure of human growth hormone in trifluoroethanol was compared to that in presence of other denaturants like urea and guanidine hydrochloride. Trifluoroethanol was found to be stabilizing the secondary structure and destabilizing the tertiary structure of protein. Finally, it was observed that trifluoroethanol can be used to solubilize inclusion bodies of a number of proteins.

**Conclusions:**

Trifluoroethanol was found to be a suitable mild solubilization agent for bacterial inclusion bodies. Fully functional, bioactive human growth hormone was recovered in high yield from inclusion bodies using trifluoroethanol based solubilization buffer. It was also observed that trifluoroethanol has potential to solubilize inclusion bodies of different proteins.

**Electronic supplementary material:**

The online version of this article (doi:10.1186/s12934-016-0504-9) contains supplementary material, which is available to authorized users.

## Background

*Escherichia coli* has been the host microorganism of choice for production of recombinant proteins due to a variety of reasons, the most important reasons being the extensive knowledge available on its physiology, ease of genetic manipulations and faster growth rate in controlled environment [1, 2]. Many times, overproduction of the target protein in *E. coli* leads to their accumulation in the form of aggregates called inclusion bodies (IBs). The major cause for formation of IBs is the high translational rate that keeps the protein concentration high inside the cytosol and allows the partially folded protein molecules to interact with each other to form aggregates [3, 4]. Most protocols for protein recovery from IBs include solubilization using a denaturing agent followed by refolding of solubilized protein and subsequent purification of the refolded protein [5, 6]. The formation of IBs is a boon in the way that it decreases the number of steps for protein purification, as IBs consist mostly of a single protein of interest due to the specific feature of protein aggregation [7, 8]. But at the same time, one cannot guarantee recovery of bioactive protein after solubilization and refolding procedures. This stresses on the need for development of new solubilization and refolding methods to improve the chances of recovery of bioactive protein from IBs.

Classically IBs were considered as aggregates with no biological activity. But recent advances have shown that IBs may contain some proportion of bioactive protein molecules [9, 10]. Recovery of active protein from such IBs relies on use of non-denaturing solubilization agents without the need of the refolding step. Some of the examples of non-denaturing solubilization agents include *N*-lauroyl sarcosine, dimethysulfoxide (DMSO) and low percentage (5 %) of *n*-propanol [9]. The yield depends on the proportion of properly folded active protein molecules present in IBs, which in turn depends on the expression conditions. Another method of recovering protein from IBs makes use of mild solubilization agents which preserve the existing native-like secondary structures in the solubilized state. Examples include solubilization using high pH buffers, detergents, low denaturant concentration and use of 6 M *n*-propanol [9–15]. Solubilization using mild agents help in improving the yield of bioactive protein as it helps in decreasing aggregation during refolding [16]. The possible reason for increased refolding yield comes from the fact that mild solubilization preserves the existing native-like secondary structure of protein molecules. If the secondary structures are on the refolding pathway, the chances of protein getting into aggregation pathway decrease. On the other hand if protein molecules get completely unfolded, the chances of accessing the aggregation pathway during refolding increase and results in low recovery of bioactive protein [14].

Human growth hormone (hGH) has been successfully recovered in bioactive form from IBs [14, 16–18]. hGH IBs require high concentration (6–8 M) of urea for solubilization. Guanidine hydrochloride (GdnHCl) is also an effective solubilization agent but refolding from GdnHCl denatured state leads to considerable aggregation and result in low recovery of bioactive protein. Even from the urea denatured state the yield at refolding step is about 50 % and overall recovery is 21 % [18]. Mild solubilization strategies have been shown to be effective for recovery of hGH from IBs. High pH buffer and *n*-propanol based buffer with 2 M urea have been described as mild solubilization agents for solubilization of hGH IBs with a high yield at refolding stage (about 70 %) [14, 16–18]. High refolding yield obtained in these cases was expected because of preservation of native-like secondary structures of protein in the solubilized state.

The effect of organic solvents on protein structure has long been studied [19, 20]. In general, alcohols like methanol, ethanol and trifluoroethanol (TFE) are known to stabilize secondary structure, mainly helical structure. The effect of organic solvents on the tertiary structure is more generalized and most of the solvents destabilize the tertiary structure [21–23]. Organic solvents affect the secondary and tertiary structure of proteins by increasing the polypeptide intramolecular hydrogen bonding, disrupting the hydrophobic interactions or by interacting with water molecules and influencing their hydrogen bonding potential [22].

Here we describe TFE as solubilization agent and establish its usefulness by showing recovery of bioactive hGH from IBs. Purified hGH was characterized using biophysical tools and cell proliferation assay. To understand the mechanism of inclusion body solubilization by TFE, secondary and tertiary structure of hGH in presence of TFE was investigated and compared to that in presence of other solubilization agents like urea and GdnHCl. It was also observed that solubilization of IBs with TFE is a widespread phenomenon and can be applied to many other proteins.

## Results and discussion

### Expression of recombinant hGH and IB preparation

**Fig. 1:**
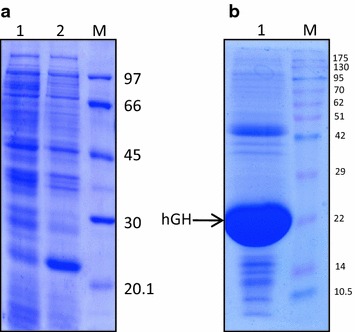
SDS-PAGE analyses of r-human growth hormone expression and IB purification. **a** Expression of r-human growth hormone. *Lane 1* uninduced cell lysate, *lane 2* induced cell lysate, *lane M* LMW marker (97, 66, 45, 30 and 20.1 kDa). **b** Isolation of IBs. *Lane 1* purified IBs, *lane M* LMW marker (175–10.5 kDa)

Recombinant hGH was expressed as 22 kDa protein in *E. coli*. About 30 % of the total cellular protein was hGH. Majority of the expressed protein was in the form of IBs. IBs were isolated by sonication followed by centrifugation. About 80 mg of IBs were obtained per liter of bacterial culture. The purity of hGH IBs was assessed by SDS-PAGE and was found to be around 90 % (Fig. 1).

### Solubilization and refolding of inclusion body protein

**Fig. 2:**
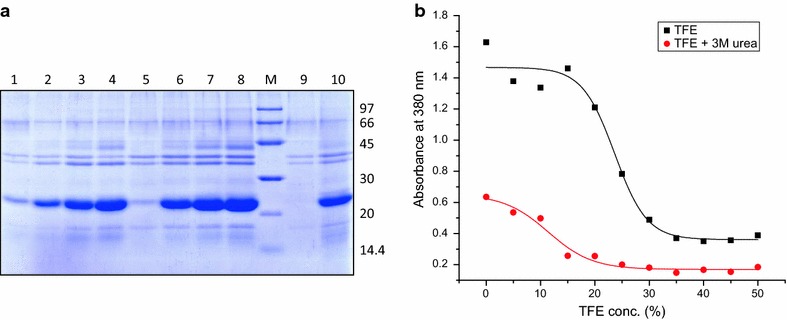
Solubilization of hGH IBs in different concentrations of TFE in presence or absence of 3 M urea. **a** SDS-PAGE analysis of hGH IB solubilization. *Lane 1*–*4* supernatant of hGH IBs solubilized in TFE concentrations ranging from 20 to 50 %, *lane 5*–*8* supernatant of hGH IBs solubilized in TFE concentrations ranging from 10 to 40 % along with 3 M urea, *lane 9* supernatant of hGH IBs solubilized in 3 M urea, *lane 10* supernatant of hGH IBs solubilized in 8 M urea, *lane M* LMW marker (97, 66, 45, 30, 20.1, 14.4 kDa). **b** Turbidimetry of hGH IBs solubilized in different concentrations of TFE in absence and presence of 3 M urea at 380 nm

The solubilization potential of TFE containing buffers was compared to those containing TFE in combination with 3 M urea (Fig. 2). Low concentration of urea (3 M) alone did not bring about appreciable solubilization of hGH IBs. TFE by itself is not a very potent solubilization agent and did not bring out complete solubilization of hGH IBs even at 50 % concentration. But use of 3 M urea with buffers containing TFE improved their solubilization potential and decreased the requirement of TFE. Complete solubilization of hGH IBs was achieved in buffer containing 30 % TFE in presence of 3 M urea (Fig. 2).

hGH IBs obtained from 1 l of bacterial culture were solubilized in buffer containing 50 mM Tris–HCl, 30 % TFE, 3 M urea and 1 mM DTT. For comparison of refolding yield, IBs obtained from 1 l of bacterial culture were also solubilized in buffer containing 50 mM Tris–HCl, pH 12, 2 M urea and 1 mM DTT (Table 1). The refolding of solubilized protein obtained by two methods was carried out under identical conditions, using the same refolding buffer. The refolding yield was calculated for both the conditions and it was observed that a higher yield is obtained when TFE based buffer is used as solubilization agent as compared to high pH buffer with 2 M urea (Table 1). About 45 % of recombinant hGH was recovered from IBs using TFE as solubilization agent (Table 2). The reason for the high refolding yield in case of TFE based solubilization could stem from partial stabilization of protein secondary structures in the solubilized state.

**Table 1:** Comparative analysis of solubilization efficiency and refolding yield of hGH from IBs while using high pH or TFE based buffer for solubilization

Solubilization buffer	Solubilized protein (mg)	Refolded protein (mg)	Solubilization efficiency (%)	Refolding yield (%)
High pH buffer with 2 M urea and 1 mM DTT	62.99 ± 3.92	34.45 ± 2.2	79.63 ± 4.96	54.89 ± 5.9
30 % TFE with 3 M urea and 1 mM DTT	71.78 ± 2.94	61.43 ± 2.97	90.75 ± 3.72	85.59 ± 2.46

**Table 2:** Purification of hGH from inclusion bodies using TFE as solubilization agent

Sample	Amount (mg)	Step yield (%)	Overall yield (%)
IBs	79.14	–	–
Solubilized protein	74.21	94	94
Refolded protein	64.83	87	82
DEAE purified protein	46.8	72	59
SEC purified protein	35.6	76	45

Details of protein yield at each step of purification and step yield indicate the suitability of this current method for high recovery of protein from inclusion bodies of *E. coli*

### Purification of hGH

**Fig. 3:**
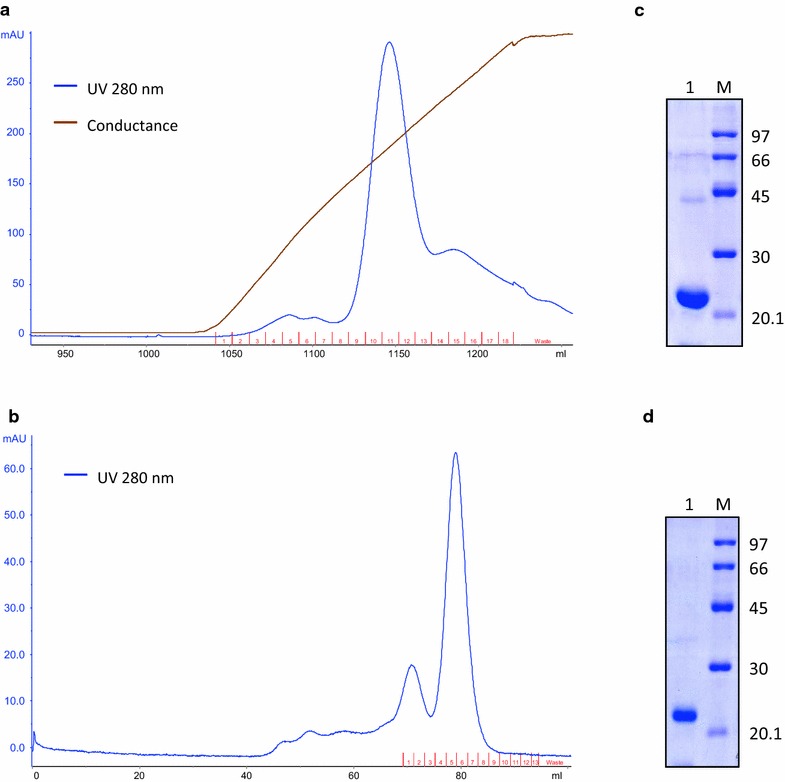
Purification of r-hGH. **a** FPLC chromatogram of r-hGH purification by DEAE-Sepharose ion-exchange chromatography. *Blue line* UV absorbance at 280 nm, *brown line* conductance. **b** FPLC chromatogram of r-hGH purification by size exclusion chromatography. **c** SDS-PAGE analysis of purified protein pooled after DEAE-Sepharose ion exchange chromatography. *Lane 1* purified r-hGH, *lane M* LMW marker (97, 66, 45, 30 and 20.1 kDa). **d** SDS-PAGE analysis of purified protein pooled after size exclusion chromatography. *Lane 1* purified r-hGH, *lane M* LMW marker (97, 66, 45, 30 and 20.1 kDa)

Refolded hGH obtained after solubilization in TFE based buffer was purified using anion exchange chromatography followed by size exclusion chromatography (SEC) (Fig. 3a, b). Recombinant hGH with purity levels more than 95 % was obtained by chromatographic methods. About 36 mg of pure hGH could be recovered from 79 mg IBs when TFE based solubilization was used (Table 2). The purity of hGH obtained after each round of purification was compared using SDS-PAGE (Fig. 3c, d).

Earlier attempts at purifying hGH from IBs using mild solubilization agents involved use of high pH buffer and *n*-propanol based buffer with 2 M urea [14, 16–18]. 8 M urea or 6 M GdnHCl have also been used as solubilization agents. A comparative study showing overall recovery of bioactive hGH from IBs using different solubilization agents has been shown in Table 3. Overall recovery of bioactive protein depends largely on refolding yield. Refolding yield obtained with mild solubilization methods is generally higher than that obtained with 8 M urea and 6 M GdnHCl. Bioactivity of the hGH refolded from IBs solubilized with mild solubilization agents were more or less similar. The improved refolding yield reflects on the overall recovery and high amount of bioactive protein could be recovered using mild solubilization methods.

**Table 3:** Comparison of different solubilization agents for recovery of hGH from IBs

Solubilization agent	Refolding yield (%)	Overall recovery (%)	References
50 mM Tris, pH 8.5, 8 M urea	50	21	[14, 18]
50 mM Tris, pH 12, 2 M urea	86	42	[17, this study]
50 mM Tris, pH 8.5, 6 M *n*-propanol, 2 M urea	76	40	[14]
50 mM Tris, pH 8.5, 30 % TFE, 3 M urea	87	45	This study

### Characterization of purified protein

**Fig. 4:**
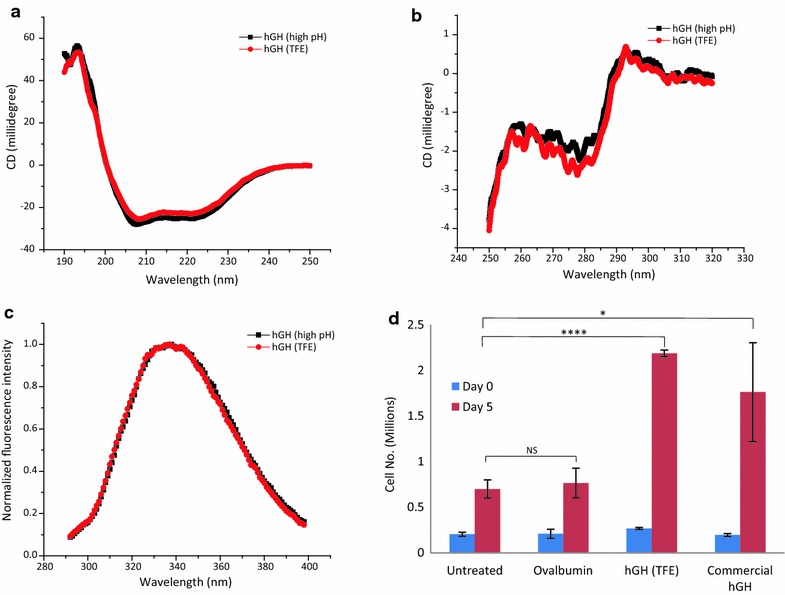
Biophysical characterization of r-hGH. **a** Far UV CD spectra of r-hGH purified after solubilization in TFE based buffer along with that of hGH purified after solubilization in high pH buffer containing 2 M urea. **b** Near UV CD spectra of r-hGH purified after solubilization in TFE based buffer along with that of hGH purified after solubilization in high pH buffer containing 2 M urea. **c** Fluorescence spectra of r-hGH purified after solubilization in TFE based buffer along with that of hGH purified after solubilization in high pH buffer containing 2 M urea. **d** Nb2 cell proliferation assay of r-hGH purified after solubilization in TFE based buffer along with that of commercial hGH. Ovalbumin (ova) was used as negative control

Purified hGH was characterized using different spectroscopic techniques. hGH obtained from IBs using high pH solubilization followed by refolding and purification was used as positive control. It has been shown previously that bioactive hGH can be recovered from IBs using high pH solubilization buffer containing 2 M urea [17, 18, 24]. Secondary structure of purified hGH as determined by far UV CD spectroscopy is shown in Fig. 4a. hGH is a completely α-helical protein with eight helices. Purified hGH showed negative bands at 208 and 222 nm, characteristic of α-helical proteins. The CD spectrum obtained was similar to that reported earlier for recombinant hGH obtained after solubilization in high pH buffer as well as native hGH [17, 18]. This also emphasizes that refolded protein obtained after solubilization in TFE based buffer was properly folded and have α-helical structures.

Tertiary structure of refolded hGH was analyzed by near UV CD (Fig. 4b) and fluorescence spectroscopy (Fig. 4c). Near UV CD spectra of purified hGH showed a positive band with peak at 292 nm and a negative band with peak at 280 nm, which suggests the formation of tertiary contacts around tryptophan and tyrosine residues respectively, emphasizing formation of proper conformations during refolding procedure. hGH purified after solubilization in high pH buffer also showed similar spectrum. Near UV CD spectra of two purified protein preparations were also similar to that of native hGH reported earlier [24]. Intrinsic tryptophan fluorescence spectra of two hGH preparations were comparable to each other and to that of native hGH [24]. hGH contains eight tyrosine residues and single tryptophan residue. To assess proper folding of hGH, protein samples were excited at 280 nm, at which both tryptophan and tyrosine absorb. Emission spectrum of tyrosine is blue shifted in comparison to that of tryptophan. Figure 4c shows fluorescence emission spectra of hGH with maxima at 337 nm. This suggested the presence of tryptophan residue buried in the hydrophobic core as exposed tryptophan shows maxima at higher wavelength. Also there is no contribution from tyrosine emission due to energy transfer from tyrosine to tryptophan in compactly folded protein. This suggests compact and properly folded structure of purified hGH obtained from IBs using TFE as solubilization agent.

### Bioactivity of purified hGH

Bioactivity of hGH was assessed using proliferation of Nb2 cell line. Nb2 cells are rat lymphoma dependent on prolactin for proliferation. It has prolactin receptors on cell surface that internalizes on prolactin binding and induces mitogenesis. hGH binding to prolactin receptor also induces cell proliferation [25]. Increase in cell number can thus be used as an assay for bioactivity of hGH. Figure 4d shows bioactivity of purified hGH. Commercial hGH (Boehringer Mannheim, Germany) was used as positive control. Untreated cells and cells treated with an unrelated protein ovalbumin are also taken as negative controls. Purified hGH showed comparable proliferation of cells at a concentration of 5 ng/ml and the proliferation was significantly higher than in control groups (p < 0.05). This proves that hGH recovered from IBs using TFE based solubilization was fully functional and biologically active.

### Solubilization of different IB proteins using TFE

**Fig. 5:**
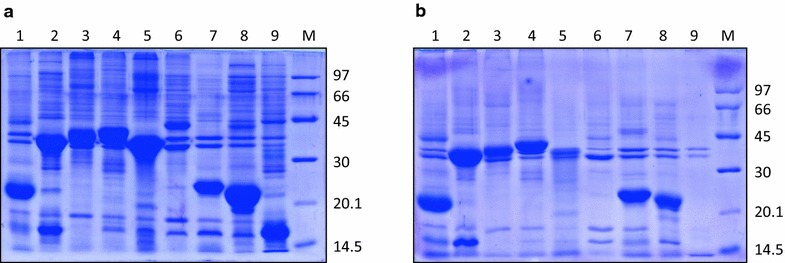
Expression of IBs of different proteins and their solubilization by TFE based buffer. **a** Expression of IBs of different proteins. *Lane 1*–*9* is hGH, enolase, asparaginase, polyketide synthase, aldolase, ovalbumin, superoxide dismutase, ovine growth hormone and lysozyme IBs respectively, *lane M* LMW marker (97, 66, 45, 30, 20.1 and 14.5 kDa). **b** SDS-PAGE analysis of IB solubilization by TFE based buffer. *Lane 1*–*9* are supernatant of hGH, enolase, asparaginase, polyketide synthase, aldolase, ovalbumin, superoxide dismutase, ovine growth hormone and lysozyme IBs solubilized in TFE based buffer respectively, *lane M* LMW marker (97, 66, 45, 30, 20.1 and 14.5 kDa)

To establish the effectiveness of TFE as solubilization agent, IBs of nine different proteins (hGH, enolase, asparaginase, polyketide synthase, aldolase, ovalbumin, superoxide dismutase, ovine growth hormone and lysozyme) were solubilized using TFE. Protein concentrations of all nine IB proteins were kept constant (40–50 mg/ml) and solubilized in fixed concentration of TFE (50 %) without addition of urea. Some of the IBs included in this study are soft IBs, i.e. they get solubilized in low concentration of urea (3 M). To keep the conditions of solubilization constant, all IBs were solubilized in buffer containing fixed concentration of TFE without urea. IBs of nine different proteins are presented in Fig. 5a. Supernatants of solubilized IBs were analyzed by SDS-PAGE and presented in Fig. 5b. Except aldolase, ovalbumin and lysozyme IBs, other IBs were efficiently solubilized using TFE. This indicated that TFE can be used to solubilize IB aggregates of many proteins.

### Structural analysis of solubilized hGH

To understand the mechanism of solubilization by TFE, its effect on secondary and tertiary structure of hGH was investigated and compared with other solubilization agents like urea and GdnHCl. The effect of different solubilization agents on secondary structure of hGH was studied using far UV CD spectroscopy and that on tertiary structure of hGH was investigated by SEC and intrinsic tryptophan fluorescence. Fluorescence spectra of hGH incubated in different buffers were also compared to fluorescence spectra of tryptophan analogue, *N*-acetyl-l-tryptophanamide (NATA) in respective buffers. Acrylamide quenching was also used as a tool to measure tryptophan accessibility of hGH in presence of different solubilization buffers.

#### Far UV CD spectroscopy of solubilized hGH

**Fig. 6:**
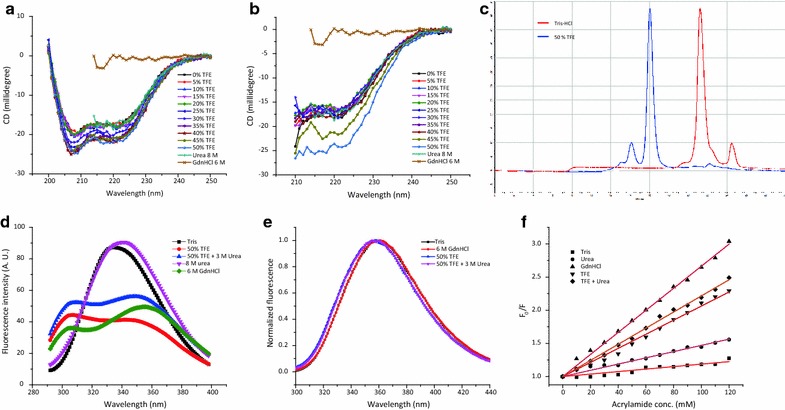
Analysis of secondary and tertiary structure of hGH in solubilized state. **a** Far UV CD spectra of hGH in 8 M urea, 6 M GdnHCl and varying concentrations of TFE. **b** Far UV CD spectra of hGH in 8 M urea, 6 M GdnHCl and varying concentrations of TFE in presence of 3 M urea. **c** Size exclusion chromatogram of hGH incubated in Tris–HCl and 50 % TFE. **d** Fluorescence spectra of hGH incubated in different buffers. **e** Normalized fluorescence spectra of NATA incubated in different buffers. **f** Stern–Volmer plots for acrylamide quenching of hGH in presence of different buffers

Secondary structure of hGH in presence of different solubilization buffers (Table 4) was investigated by far UV CD spectroscopy and is presented in Fig. 6a, b. hGH is a completely α-helical protein with eight helices. Its far UV CD spectrum is characterized by negative bands at 208 and 222 nm. Figure 6a shows the effect of different concentrations of TFE on secondary structure of hGH and compares it with that observed with 8 M urea or 6 M GdnHCl. Similarly Fig. 6b shows the effect of different concentrations of TFE in combination with 3 M urea on hGH secondary structure. It was observed that increasing concentrations of TFE had a stabilizing effect on secondary structure of hGH. At concentrations above 25 % of TFE, there was an increase in ellipticity at 208 and 222 nm indicating stabilization of α-helical structure. Addition of 3 M urea to different concentrations of TFE also had the same effect on secondary structure, although the magnitude of stabilization was lesser at lower TFE concentrations (Fig. 6b). CD spectra of hGH incubated in presence of 8 M urea showed no effect on the secondary structure indicating resistance of hGH towards denaturation by urea. CD spectra of hGH in presence of 6 M GdnHCl on the other hand, showed complete loss of helicity indicating complete loss of secondary structure. TFE based buffer thus protects the secondary structure of protein in the solubilized state.

**Table 4:** Composition of solubilization buffers used for CD spectroscopy

Buffer	Buffer composition
a	10 mM Tris–HCl, pH 8.5
b	8 M urea, 10 mM Tris–HCl, pH 8.5
c	6 M GdnHCl, 10 mM Tris–HCl, pH 8.5
d	0–50 % TFE, 10 mM Tris–HCl, pH 8.5
e	0–50 % TFE, 3 M urea, 10 mM Tris–HCl, pH 8.5

#### Size exclusion chromatography of solubilized hGH

SEC is routinely used to characterize the hydrodynamic radii and molecular weight of globular proteins. SEC is a separation based technique and works on the principle that larger sized particles elute first, followed by smaller sized particles. It is a better method to characterize the tertiary structure of globular proteins as it gives information based on global structure unlike fluorescence based techniques that gives information about the environment of the fluorophore (tryptophan or tyrosine) only. SEC was used to find out the effect of TFE based buffer on the hydrodynamic radii of hGH. Hydrodynamic radii of hGH in presence of TFE based buffer was compared to that in presence of Tris–HCl buffer. Figure 6c shows that hGH in presence of Tris–HCl buffer elutes at about 21 min and in presence of TFE based buffer elutes at about 16 min. This shows that hGH is properly folded and has a compact structure in presence of Tris–HCl buffer with a smaller hydrodynamic radii, while in presence of TFE based buffer, the protein loses its compact structure and its hydrodynamic radii increases. This suggests that TFE based buffer disrupts the tertiary structure of hGH.

#### Fluorescence spectroscopy of solubilized hGH

The tertiary structure of hGH in presence of TFE based buffer was analyzed by fluorescence spectroscopy and was compared to that in presence of other solubilization buffers (Table 5). hGH contains one tryptophan and eight tyrosine residues, which makes it a very good model for fluorescence studies. Selective information about the environment of tryptophan can be obtained by exciting at 295 nm, where tyrosine residues do not absorb. In case of hGH, excitation of both tyrosine and tryptophan residues at 280 nm is beneficial, as it helps in discriminating native state from partially folded and unfolded states. In the folded state the tyrosine residues are in vicinity of tryptophan and due to energy transfer from tyrosine to tryptophan, tyrosine emission is not observed and only a single peak of tryptophan emission is observed at 337 nm, which shows that tryptophan is buried in the hydrophobic core. In the denatured state though, tyrosine residues are separated from tryptophan residue and tyrosine emission at 305 nm is observed due to failure of energy transfer and a red shift in tryptophan emission is seen at 357 nm [24, 26].

**Table 5:** Composition of buffers used for fluorescence spectroscopy

Buffer	Buffer composition
a	50 mM Tris–HCl, pH 8.5
b	8 M urea, 50 mM Tris–HCl, pH 8.5
c	6 M GdnHCl, 50 mM Tris–HCl, pH 8.5
d	50 % TFE, 50 mM Tris–HCl, pH 8.5
e	50 % TFE, 3 M urea, 50 mM Tris–HCl, pH 8.5

Figure 6d shows fluorescence spectra of hGH in presence of Tris–HCl buffer (native protein) and in 6 M GdnHCl (denatured protein). In presence of 8 M urea, hGH spectrum looks similar to that of native protein, with a slight red shift to 342 nm. This shows that hGH is resistant to denaturation by urea. In presence of TFE based buffers, hGH spectrum looked like that of denatured protein in presence of 6 M GdnHCl. Two separate peaks, one for tyrosine emission at 300 nm and other for tryptophan emission at 352 nm were observed. Tryptophan emission was slightly blue shifted as compared to that in presence of GdnHCl that points to the possibility that hGH structure in presence of TFE based buffers is not that opened up as in case of 6 M GdnHCl. Another possibility is that hGH structure in TFE based buffers is as opened up as in case of 6 M GdnHCl and the slight blue shift of 5 nm in case of TFE based buffers could be due to solvent effects, as polarity of TFE is lesser as compared to water. To find out the effect of solvent polarity on emission maxima, fluorescence properties of tryptophan analogue, NATA were studied in presence of different buffers (Table 6). Figure 6e shows NATA fluorescence in different buffers. Emission maxima of NATA in presence of TFE based buffers were slightly blue shifted as compared to that in presence of Tris–HCl and 6 M GdnHCl. This suggests that the protein tertiary structure in presence of TFE based buffers is as denatured as in case of 6 M GdnHCl.

**Table 6:** Composition of buffers used for NATA fluorescence

Buffer	Buffer composition
a	50 mM Tris–HCl, pH 8.5
b	6 M GdnHCl, 50 mM Tris–HCl, pH 8.5
c	50 % TFE, 50 mM Tris–HCl, pH 8.5
d	50 % TFE, 3 M urea, 50 mM Tris–HCl, pH 8.5

#### Tryptophan accessibility of hGH in different solubilization buffers by acrylamide quenching

To further understand the effect of TFE based buffers on structure of hGH, acrylamide quenching studies were carried out. Acrylamide is a collisional quencher and decreases the fluorescence intensity by interacting with the fluorophore and promoting non-radiative transfer to the ground state. Acrylamide quenching is used to find out the accessibility of tryptophan in a protein molecule. If tryptophan residue is buried in the hydrophobic core of a compactly folded protein molecule, increasing concentrations of acrylamide, do not bring out a big decrease in the fluorescence intensity. On the other hand, if a tryptophan residue is surface exposed or in denatured protein, where tryptophan is solvent exposed, increasing concentrations of acrylamide substantially decrease the fluorescence intensity. The decrease in fluorescence intensity with quencher concentration is related with a mathematical equation known as Stern–Volmer equation (Eq. 1).

In this study, hGH was incubated in presence of different buffers (Table 7) and tryptophan accessibility in each case was measured using acrylamide quenching. Figure 6f shows Stern–Volmer plots obtained after acrylamide quenching experiments with hGH incubated in five different buffers. Table 8 shows Stern–Volmer quenching constants obtained after fitting the data in Eq. 1. It was concluded from Fig. 6f and Table 8 that hGH incubated in Tris–HCl buffer shows minimum change in fluorescence intensity and has least value of Stern–Volmer quenching constant (0.19 × 10^−2^). This shows that tryptophan is shielded from the solvent and has least accessibility in Tris–HCl buffer. Maximum accessibility of tryptophan can be seen in case of GdnHCl buffer with Stern–Volmer quenching constant of 1.66 × 10^−2^. This is expected as it is widely reported that 6 M GdnHCl completely denatures hGH. hGH is known to be resistant to denaturation by urea. Stern–Volmer quenching constant obtained in case of urea reflects that with a value of 0.44 × 10^−2^. Stern–Volmer quenching constants obtained in case of TFE based buffers have values slightly lesser than that observed with GdnHCl. This points to the possibility that hGH is not completely denatured by TFE based buffers. But as already seen in case of intrinsic tryptophan fluorescence experiments, this could also be due to the solvent effects i.e. decreased polarity of TFE in comparison to water. In Fact, TFE, because of presence of halogen groups act as a quencher and NATA fluorescence in presence of TFE based buffers is lesser as compared to that in presence of Tris buffer, pH 8.5 (Additional file 1: Figure S1a). Acrylamide quenching experiment with NATA also shows lesser slope with TFE based buffers than GdnHCl and Tris buffer (Additional file 1: Figure S1b).

**Table 7:** Composition of buffers used for acrylamide quenching experiments

Buffer	Buffer composition
a	50 mM Tris–HCl, pH 8.5
b	8 M urea, 50 mM Tris–HCl, pH 8.5
c	6 M GdnHCl, 50 mM Tris–HCl, pH 8.5
d	50 % TFE, 50 mM Tris–HCl, pH 8.5
e	50 % TFE, 3 M urea, 50 mM Tris–HCl, pH 8.5

**Table 8:** Stern–Volmer quenching constants for acrylamide quenching of hGH incubated in different buffers

Buffer	Stern–Volmer quenching constant
a	0.19 × 10^−2^
b	0.47 × 10^−2^
c	1.66 × 10^−2^
d	1.06 × 10^−2^
e	1.22 × 10^−2^

## Conclusions

This study establishes the effectiveness of TFE based solubilization agent for recovery of hGH from IBs with improved yield as compared to urea based solubilization agents. The overall yield of purified recombinant hGH using TFE based solubilization method is similar to already described solubilization methods using high pH buffer and *n*-propanol based buffer. It was also observed that TFE acts as a mild but effective solubilization agent by stabilizing secondary structures and destabilizing tertiary structures. None of the solubilization agents known act as universal solubilization agent. Screening of solubilization agents for every protein is thus necessary for finding efficient ways for recovering active protein from IBs [27]. As TFE is known to induce secondary structures in proteins, mainly alpha helices, it could be really helpful in solubilization of proteins that adopt an alpha helix rich native state. As most of the IB proteins have native-like secondary structure, it is expected that solubilization with TFE will protect the existing native-like secondary structure and thus will enhance the recovery of bioactive protein from solubilized state. Ability of TFE to solubilize a number of IB proteins encourage its use as solubilization agent for IB proteins that cannot be recovered by conventional methods. This study also encourages screening of different organic solvents for solubilization of IB aggregates for high throughput recovery of bioactive protein.

## Methods

### Chemicals and reagents

Components for culture media, tryptone and yeast extract were purchased from Difco Laboratories, India. Glycine, phenylmethylsulfonyl fluoride (PMSF), isopropyl β-d-1-thiogalactopyranoside (IPTG), Tris buffer and sodium dodecyl sulphate (SDS) were from Amresco, USA. Glucose and NaCl were purchased from Qualigen, India. Low molecular weight marker for SDS-PAGE and DEAE-Sepharose Fast Flow media were from GE Healthcare, USA. Dithiothreitol (DTT), acrylamide, bis-acrylamide, urea, ammonium persulphate (APS), TFE, NATA and ovalbumin were purchased from Sigma-Aldrich, USA. Bromophenol blue, Tetramethylethylenediamine (TEMED) and Ethylenediaminetetraacetic acid (EDTA) were procured from BIO-RAD, USA. Micro BCA assay kit was purchased from Pierce, USA.

### Expression of recombinant proteins and IB preparation

*Escherichia coli* BL21 (DE3) or M15 (pREP4) cells were transformed with different vectors encoding recombinant proteins (hGH, enolase, asparaginase, polyketide synthase, aldolase, ovalbumin, superoxide dismutase, ovine growth hormone and lysozyme) [28, 29]. Table 9 shows details of recombinant protein clones in *E. coli*. The transformed cells were cultured overnight in LB agar plates supplemented with ampicillin and kanamycin at 37 °C. Single colonies were picked and grown overnight in modified LB broth containing 5 g/l glucose (37 °C, 200 rpm) supplemented with either 100 μg/ml ampicillin or 100 μg/ml ampicillin and 25 μg/ml kanamycin depending on the construct. The overnight grown cultures were diluted with fresh modified LB broth containing antibiotics and allowed to grow until OD_600_ reached 0.5. The culture was then induced with 1 mM IPTG and allowed to grow for 3 h. The cells were harvested by centrifugation and checked for expression of hGH by SDS-PAGE.

**Table 9:** Details of recombinant proteins expressed as inclusion bodies

Protein	Expression host	Expression vector	Antibiotic selection pressure
Human growth hormone	*E. coli* M15 (pREP)	pQE 60	Ampicillin, kanamycin
Enolase	*E. coli* BL21 (DE3)	pET 22b	Ampicillin
Asparaginase	*E. coli* BL21 (DE3)	pET 14b	Ampicillin
Polyketide synthase	*E. coli* BL21 (DE3)	pET 22b	Ampicillin
Aldolase	*E. coli* BL21 (DE3)	pET 22b	Ampicillin
Ovalbumin	*E. coli* BL21 (DE3)	pET 3d	Ampicillin
Superoxide dismutase	*E. coli* M15 (pREP)	pQE 60	Ampicillin, kanamycin
Ovine growth hormone	*E. coli* M15 (pREP)	pQE 60	Ampicillin, kanamycin
Lysozyme	*E. coli* BL21 (DE3)	pET 21c	Ampicillin

The *E. coli* cell pellet obtained from 1 l culture was resuspended in 20 ml of 50 mM Tris–HCl, pH 8.5, 100 mM NaCl, 1 mM PMSF and 5 mM EDTA and sonicated on ice at amplitude of 50 for 10 cycles with 1 min gap between each cycle. Each cycle of 1 min comprised of alternate on and off pulses of 1 s (Q 700 sonicator, Qsonica, USA). The lysed bacterial suspension was centrifuged at 15,000*g* for 20 min at 4 °C (Sorvall RC 6+, USA). The pellet obtained was washed by resuspending in MQ water and centrifuging at 15,000*g* for 20 min at 4 °C. The washing process was repeated twice. The washed IBs were finally resuspended in 4 ml of MQ water.

### Solubilization of IBs and refolding of solubilized protein

The IBs obtained were solubilized in different concentrations of TFE ranging from 5 to 50 % in order to check its solubilization potential. Ten microlitre of IB suspension was incubated in 90 μl buffers containing 50 mM Tris–HCl, pH 8.5 and varying concentrations of TFE (5–50 %) at room temperature for 2 h. The turbidity of the samples was then monitored at 350 nm to determine the solubilization potential. Similarly, the solubilization potential of buffers containing different concentrations of TFE (5–50 %) along with low concentration of urea (3 M) was also determined. Solubilization potential of different buffers was also checked by SDS-PAGE. The solubilized samples (20–50 % TFE and 10–40 % TFE with 3 M urea) were centrifuged at 15,000*g* for 10 min at 25 °C. Equal volume (10 μl) of supernatant was loaded on SDS-PAGE to compare solubilization potential of various solubilization buffers.

The IBs obtained from 1 l of bacterial culture were solubilized in 25 ml of solubilization buffer containing 50 mM Tris–HCl, pH 8.5, 3 M urea, 30 % TFE and 1 mM DTT. The suspension was incubated at room temperature for 2 h and then centrifuged at 15,000*g* for 30 min. The supernatant was added into 250 ml of refolding buffer containing 50 mM Tris–HCl, pH 8.5. The refolded sample was centrifuged at 24,000*g* for 30 min at 4 °C to obtain the supernatant. Protein concentration in supernatant, measured by BCA method, was compared to that in solubilized sample to calculate the refolding yield. The supernatant was then used for chromatographic purification. For comparison, IBs obtained from 1 l bacterial culture were also solubilized in 25 ml of solubilization buffer containing 50 mM Tris–HCl, pH 8.5, 8 M urea and 1 mM DTT. The suspension was processed in the same manner as mentioned above to obtain refolded protein and for calculation of refolding yield.

### Purification of hGH

Refolded hGH was purified using ion exchange and SEC. DEAE-Sepharose Fast Flow media (40 ml) was packed in XK-26/40 column (Pharmacia, Sweden). Column was washed with 250 ml MQ water and equilibrated with 200 ml of equilibration buffer (50 mM Tris, pH 8.5). Refolded r-hGH (250 ml) was loaded onto DEAE-Sepharose column and washed with 150 ml of equilibration buffer. Elution was carried out using a gradient of NaCl ranging from 0 to 500 mM. Equilibration, protein loading, washing and elution were performed at a flow rate of 5 ml/min. Homogeneity of eluted protein was checked by SDS-PAGE.

Eluates containing hGH were pooled and dialyzed against 50 mM Tris–HCl, pH 8.5. Dialyzed sample was concentrated to 2 ml using Amicon Ultra centrifugal concentrator (Millipore, USA) with molecular weight cutoff 3 kDa. Concentrated sample was centrifuged at 15,000*g*, 4 °C for 10 min. Two ml of concentrated sample was loaded for SEC onto HiLoad 16/600 Superdex 200 PG column (GE healthcare, UK) pre-equilibrated with 250 ml of 50 mM Tris–HCl, pH 8.5 at a flow rate of 1 ml/min. Homogeneity of eluted protein was checked by SDS-PAGE. The molar extinction coefficient of hGH was taken as 18,890/M/cm as reported earlier [30] and was used to calculate the concentration of pure hGH. Protein concentration of the IBs, solubilized protein and refolded protein was determined by BCA assay kit (Pierce, USA) using BSA as standard.

### Fluorescence and circular dichroism (CD) spectroscopy of recombinant hGH

Fluorescence emission spectra of purified recombinant hGH were recorded using the Cary Eclipse spectrofluorimeter (Varian, USA). 50 µg/ml solution of purified hGH in buffer containing 50 mM Tris–HCl, pH 8.5 was excited at 280 nm and emission spectra were collected from 290 to 400 nm with excitation and emission slit width set at 5 nm. Far UV circular dichroism (CD) spectra of purified recombinant hGH were recorded using Jasco-700 spectro-polarimeter in the wavelength range of 200–250 nm at 25 °C. A 200 µg/ml solution of purified recombinant hGH in buffer containing 20 mM Tris–HCl, pH 8.5 was taken in 1 mm path length cuvette. Near UV CD spectra of purified protein were recorded in the wavelength range 250–300 nm at 25 °C. One mg/ml solution of purified hGH in the aforementioned buffer was taken in 10 mm pathlength cuvette. Each spectrum was scanned three times and the average spectrum was plotted.

### Cell proliferation assay using Nb2 cells

Nb2 cell line was maintained in RPMI medium containing 10 % FCS and 10 % HS. The cells were arrested in G_0_/G_1_ phase by incubating in RPMI medium in presence of 1 % FCS and 10 % HS. The cell proliferation assay was performed in a flat bottom 96 well plate. Commercial hGH (Boehringer Mannheim, Germany) was used as positive control [17]. An unrelated protein, ovalbumin was taken as negative control. The cellular proliferation was checked by counting the number of cells at definite intervals after incubating the cells in presence of 5 ng/ml of purified hGH. Statistical significance of the results was analyzed by unpaired t test (p < 0.05) in the GraphPad Prism software version 6.

### Far UV CD spectroscopy of hGH in solubilized state

Far UV CD spectra of hGH were recorded using Jasco-700 spectro-polarimeter in the wavelength range of 200–250 nm at 25 °C. 200 µg/ml solution of purified hGH incubated in different buffers (Table 4) for 6 h were taken in 1 mm path length cuvette. An average of three independent spectra acquired for each sample was used for analysis.

### Size exclusion chromatography of hGH in solubilized state

In this study, hGH was incubated in either 50 mM Tris–HCl, pH 8.5 or 50 mM Tris–HCl, pH 8.5 containing 50 % TFE. 10 µl of hGH samples incubated in different buffers were loaded onto the TSKgel G3000SW_XL_ column (Tosoh, Japan) with internal diameter 4.6 mm and length 30 cm pre equilibrated with 50 ml of respective buffers at a flow rate of 0.5 ml/min. Elution profile of protein was followed by monitoring UV absorbance at 280 nm on Shimadzu UFLC system with CBM-20A system controller.

### Fluorescence spectroscopy of hGH in solubilized state

Fluorescence emission spectra were recorded using the Cary Eclipse spectrofluorimeter (Varian, USA). 100 µg/ml solution of purified hGH or 5 µM solution of NATA incubated in different buffers (Tables 5, 6) for 6 h were excited at 280 nm and emission spectra were collected from 290 to 400 or 300 to 440 nm with excitation and emission slit width set at 5 nm. An average of three independent spectra acquired for each sample was used for analysis.

### Tryptophan accessibility of hGH in different solubilization buffers by acrylamide quenching

For this experiment purified hGH and NATA were incubated in different buffers (Table 7) for 6 h at room temperature. Final concentration of hGH used was 200 µg/ml. 1 ml of hGH solution in respective buffer was taken in a 10 × 4 mm cuvette and 5 M acrylamide solution was added to achieve acrylamide concentrations ranging from 0 to 120 mM. Samples were incubated for 30 min. Samples were then excited at 295 nm and emission spectra were acquired in the range of 300–400 nm. Excitation and emission slit width were set at 5 nm. Each spectrum was scanned three times and the average spectrum was plotted. Stern–Volmer plots were generated by plotting relative fluorescence intensities against acryamide concentrations and data were fitted into Stern–Volmer equation (Eq. 1) to obtain Stern–Volmer quenching constants.

1 $${\text{F}}_{0} /{\text{F}} = 1 + {\text{K}}[{\text{Q}}]$$

F_0_ is steady state fluorescence intensity in absence of quencher, F is steady state fluorescence intensity in presence of quencher, Q is quencher concentration and K is Stern–Volmer quenching constant which is a measure of exposure of the fluorophore to the solvent.

## Additional file

10.1186/s12934-016-0504-9 Analysis of NATA fluorescence and acrylamide quenching of NATA. a) Fluorescence spectra of NATA incubated in different buffers. b) Stern–Volmer plots for acrylamide quenching of NATA in presence of different buffers.

## Abbreviations

APS: ammonium persulfate
BCA: bicinchoninic acid
BSA: bovine serum albumin
DMSO: dimethylsulfoxide
DTT: dithiothreitol
EDTA: ethylenediaminetetraacetic acid
GdnHCl: guanidine hydrochloride
hGH: human growth hormone
IB: inclusion body
IPTG: isopropyl β-d-1-thiogalactopyranoside
NATA: *N*-acetyl-l-tryptophanamide
PMSF: phenylmethanesulfonyl fluoride
SDS-PAGE: sodium dodecyl sulfate polyacrylamide gel electrophoresis
SEC: size exclusion chromatography
TEMED: tetramethylethylenediamine
TFE: trifluoroethanol
