# Supplementary figures and images for: Recovery of bioactive protein from bacterial inclusion bodies using trifluoroethanol as solubilization agent
Source: Microb Cell Fact. 2016 Jun 8;15:100. doi: 10.1186/s12934-016-0504-9 (PMC4898390; doi:10.1186/s12934-016-0504-9)

## Slide 1
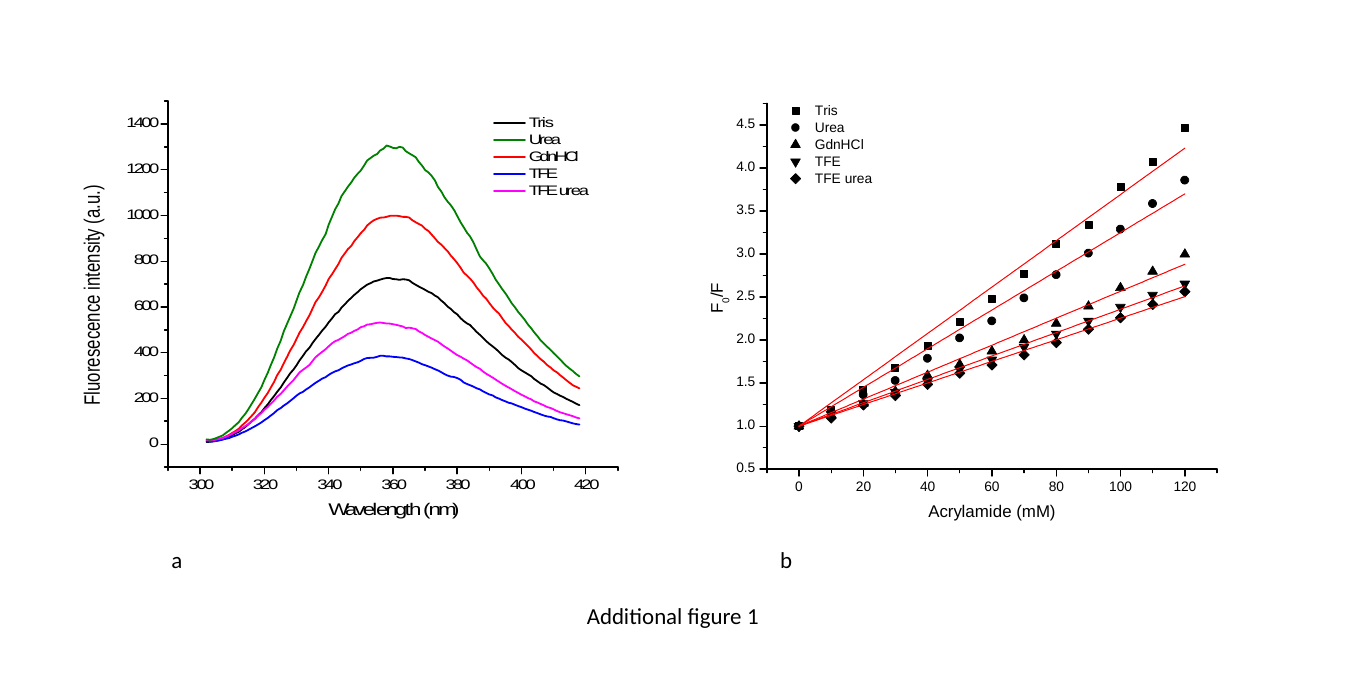

a
b
Additional figure 1

Supplement: Supplementary file 1 — 10.1186/s12934-016-0504-9 Analysis of NATA fluorescence and acrylamide quenching of NATA. a) Fluorescence spectra of NATA incubated in different buffers. b) Stern–Volmer plots for acrylamide quenching of NATA in presence of different buffers. [file 12934_2016_504_MOESM1_ESM.pptx]
